# Positive Influences of Social Support on Sense of Community, Life Satisfaction and the Health of Immigrants in Spain

**DOI:** 10.3389/fpsyg.2019.02555

**Published:** 2019-11-15

**Authors:** Isabel Hombrados-Mendieta, Mario Millán-Franco, Luis Gómez-Jacinto, Felipe Gonzalez-Castro, María José Martos-Méndez, Alba García-Cid

**Affiliations:** ^1^Department of Social Psychology, University of Málaga, Málaga, Spain; ^2^Facultad de Psicología, Universidad de Málaga, Málaga, Spain; ^3^Faculty of Social and Labour Studies, University of Málaga, Málaga, Spain; ^4^Center for Health Promotion and Disease Prevention, Arizona State University, Phoenix, AZ, United States

**Keywords:** immigrants, social support, sense of community, satisfaction with life, mental health problems, physical illness

## Abstract

The main objective of this study was to investigate the association of social support and the sense of community (SOC) with satisfaction with life (SWL) and immigrant health. We propose a model in which perceived social support from close sources (family and friends), as mediated by SOC and life satisfaction, would be positively associated with mental and physical health. Limited evidence exists from multivariate models that concurrently examine the association of both factors with SWL and health-related outcomes. We investigate the hypothesized association in a structural equations model (SEM) analysis. The participants consisted of 1131 immigrants (49% men and 51% women) (age 18–70, *M* = 33). The study was conducted in Malaga (Spain). Cross-sectional data were collected using a random-route sampling and survey methodology. In this model, greater social support from native friends was associated with a greater SOC. Social support from family and native friends was associated with greater SWL. Also, a greater SOC was associated with greater SWL. No association was found between SOC and mental health symptoms, whereas, greater SWL was associated with fewer mental health and illness symptoms. These results suggest that among immigrants, support networks involving family and native friends, and integration into the community are important influences for immigrants to achieve life satisfaction. These results are widely applicable and have implications that are relevant to the design of health promotion interventions.

## Introduction

Migration to new environments in search of better life opportunities is a pervasive phenomenon that occurs worldwide, and with significant variations within various regions of the world. In the process of migration, migrants settle in local communities or neighborhoods, many of which consist of diverse multicultural environments. An immigrants’ reception and their process of integration into a new community constitute important factors that affect migrants’ health and well-being (Chen et al., 2017; Giacco et al., 2018; Rapp et al., 2018; Vitale and Doherty, 2018; Brydsten et al., 2019; Millán-Franco et al., 2019). This process of adjustment is complex and often stressful because of the multitude of changes and various losses that migrants experience (Sutherland, 2007).

According to González and Requena (2005) during the past decade, there has been a steady increase in number of migrants coming to Spain. From 2000 to the present, Spain has emerged as a prioritized host country for migration from Africa, Latin America, Asia, and Eastern Europe. Spain, and specifically the city of Malaga, is an attractive multicultural setting for migration based on its geographical location as a “gateway to Africa,” its links with Latin America, and its employment opportunities for working within the tourism industry (Perniìa and Narvaìez, 2003). In Spain, the rights and citizenship of migrants are subject to work and residence permits along with compliance with a several requirements (Albertiìn, 2016). The recent global economic crisis has significantly affected vulnerable immigrants from Spanish territories, who have been exposed various forms of discrimination. In light of worldwide socio-political changes that affect the plight of immigrants, further study is needed to obtain a better understanding of current effects of this process of international migration, integration, and social adaptation, as they affect the health and well-being of immigrants (Hildegard, 2012).

The study of stressors and adaptation to new environments is important to identify the most salient factors that affect the physical and mental health of diverse cohorts of international immigrants. In particular, migration to distant and culturally different environments often introduces stressors that fragment and can destabilize the nuclear family. With this social displacement and separation from family (Hao and Johnson, 2000; Malone and Dooley, 2006), along with exposures to stressful and often dangerous situations and environments, migrant populations often exhibit high prevalence rates of psychopathological disorders, primarily involving anxiety, depression, and somatization (Zarza and Sobrino, 2007). Mental health and well-being are often affected by difficult social conditions that include economic problems encountered during migration (Tunstall et al., 2015). Thus, for understanding the well-being of migrants the effects of economic and social insufficiencies should be examined for their effects on immigrants’ physical and mental health (Skaff et al., 2003).

As noted, international immigrants often face highly stressful challenges within a new settlement community, thus increasing their risks of developing mental and physical disorders (Bhugra, 2004; Font et al., 2012; Bak-Klimek et al., 2015; Garcini et al., 2016). Immigrants, who experience chronic and elevated levels of stress are at greater risks of developing diagnosable psychiatric disorders, including anxiety and depressive disorders, as well as post-traumatic stress disorder (PTSD) (Buchegger-Traxler and Sirsch, 2012; Bamishigbin et al., 2017; Thibeault et al., 2018; Calzada and Sales, 2019).

Regarding physical health, some studies have clearly shown that immigrants exhibit lower levels of health when compared with natives (Jonnalagadda and Diwan, 2005). In particular, immigrants may suffer from headaches and symptoms of exhaustion, which have been interpreted as physical responses to the cognitive overload resulting from migration (Kirkcaldy et al., 2005). Cumulative exposures to chronic stress with greater time of residency within a new environment have been shown to contribute to a deterioration in health among immigrants adjusting to new living conditions within their settlement community (McClure et al., 2015; Prapas and Mavreas, 2016).

Studies have shown that the great number changes involved in the process of migration are associated with higher levels of stress. In turn, these stress levels are associated with the onset of physical and mental disorders, particularly when exacerbated by the lack of social supports and community integration (Xu and Chi, 2013; Tunstall et al., 2015).

Studies on the social integration and well-being of immigrants have thus focused on indicators of subjective well-being (McMichael and Manderson, 2004; Amit, 2010). Support supports constitute human resources that appear to serve as an important factor that affects immigrants’ well-being (Kristiansen et al., 2006), their sense of community (SOC), and their life satisfaction (Sonn, 2002; Hombrados-Mendieta et al., 2013b). Community integration is associated with effective interactions with local community residents, and with the development of local sources of social support. In turn, among immigrants, each of these may constitute resource factors that are associated with high life satisfaction (Safi, 2010; Carpentier and de la Sablonnière, 2013).

Nonetheless, few studies have examined an integrated model of the role of social support and the SOC as protective factors that can promote immigrant well-being and health. In addition, few studies have taken a multidimensional approach in the assessment of social supports, with the aim of identifying the distinct sources of support that are most strongly associated with the well-being of immigrants. Accordingly, the main objective of the present study was to investigate the systemic association between social support, the SOC, and immigrant satisfaction with life (SWL) and health.

We propose a model in which perceived social support from close sources, as mediated by the SOC and life satisfaction, would be positively associated with mental and physical health. In the past, multivariate models that have examined the association of both of these factors on life satisfaction and health-related outcomes have yielded limited results. The present study aims to clarity the association between types of social support and the SOC, and their effect on life satisfaction, mental health problems, and physical illness within an international immigrant population.

## Social Support, Well-Being and Health of Immigrant Populations

Social support is a complex multidimensional construct (Lynch, 1998). Having social support appears important for international immigrants as social capital that can facilitate their adaptive adjustment within a new community also aiding in the development of the SOC within the new community environment (Hernaìndez et al., 2005). There are many definitions of social support, with one of the most comprehensive provided by Lin et al. (1986). They describe social support as the provision of *real* and *perceived* support, both *instrumental* and *expressive*, as received from the *community*, *social networks*, and *close friends*. Most authors identify three types of social support: *emotional support*, which refers to the feeling of being loved and the security of being able to trust someone; *instrumental support*, which refers to having direct help available; and *informational support*, which consists in the provision of advice or guidance (Wills and Fegan, 2001; Taylor, 2006; Wong et al., 2007). These dimensions describe important resources that can satisfy specific needs, while promoting a sense of wellness.

The assertion that social support has a beneficial effect on well-being and health has received wide support within the literature (Caplan, 1974; Cassel, 1974; Cobb, 1976; House, 1981; Cohen and Syme, 1985; Vaux, 1988). Feeling loved and supported by others makes us feel good, also re-framing life’s challenges in new ways (Cutrona, 1986). Social support can also buffer the negative effects of stress (Thoits, 1982). Individuals who have greater levels of support exhibit a lower incidence, prevalence, and severity of illness, whether assessed with general health indicators or by the presence of chronic illness (Asher, 1984; Monroe and Johnson, 1992; Uchino et al., 1996). By contrast, a lack of support, isolation, and a limited social network have been associated with impaired physical and psychological health and a higher risk of death (Kennedy et al., 1990).

Social support has been shown to provide various benefits. The resources provided by others also facilitate direct action and effective coping. Social support aids in a realistic assessment of resources, allows stressors to be re-appraised as less threatening, and assists in the development of problem-solving strategies (Cohen and Syme, 1985). The presence of social networks and contact with others also appears to facilitate the development of a SOC and can provide positive experiences that influence well-being (Vaux, 1988). Integration in social networks can also promote the perception that support is available when needed, leading to greater comfort and reduction of perceived stress. In summary, positive psychological states can promote healthy behaviors that in turn can produce enhanced health outcomes (Hombrados, 2013).

Conversely, the loss of social support networks appears to be a major stressor that immigrants often experience, whereby several studies have found a positive association between social support and physical and mental health and well-being (Kung et al., 2003; Guruge et al., 2015). Highlighting the importance of social supports in well-being, international immigrants face highly stressful challenges in adapting to a new community environment, thus increasing their risks of developing mental disorders (Bak-Klimek et al., 2015). Ample evidence exists on a positive association between supportive social relationships and the mental well-being (Peirce et al., 2000; Oppedal and Røysamb, 2004; Xu and Chi, 2013). By contrast, in immigrant populations, several studies have highlighted the protective effects of social support networks in safeguarding against mental disorder (Levecque et al., 2009; Tieu and Konnert, 2014; Salinero-Fort et al., 2015). The social supports have aptly been described as a buffer against depression and anxiety (Shin, 1994; Martiìnez et al., 2001) and against schizophrenia in immigrant populations (Bhugra, 2004).

Regarding physical health, the presence of social support and effective social inclusion has been shown to be instrumental in promoting preventive and health-maintaining behaviors, in relation to: diabetes self-management (Chun et al., 2011; Njeru et al., 2016), the management of hypertension (Beune et al., 2010), and reducing the risk of cardiovascular disease (Zlotnick et al., 2015). Several studies have examined health behaviors among immigrants, such as physical activity and maintaining a healthy diet. Among immigrants, a lack of time, limited resources, and insufficient social supports, as well as immigration stressors have been identified as the major barriers to physical activity, often leading to more sedentary lifestyles, weight gain, and obesity (Nobari et al., 2013; Allen et al., 2014; Ramos et al., 2016). In addition, immigrants are often affected by poor sleep quality, leading to a deterioration in physical health (Jackson et al., 2014). Interpersonal relationships also appear as facilitators of the initiation and maintenance of several risk behaviors that include smoking, drinking, drug misuse, and their co-occurrence (Viner et al., 2006).

Studies on social support among immigrants have focused on factors that influence subjective well-being (e.g., McMichael and Manderson, 2004; Amit, 2010). Generally, a positive association has been found between social support and well-being (Davis et al., 1998; Zhou and Lin, 2016). Support resources have been identified as an important predictor of well-being (Kristiansen et al., 2006). Fernández et al. (2015) have suggested that social support operates as a protective resource for promoting well-being among immigrant populations. In general, studies have found an association between greater life satisfaction and perceived support. However, other studies have found life satisfaction to be a mediator of the effect of perceived support on psychological adjustment (e.g., Birman et al., 2014). Further, several studies have found that life satisfaction mediates the relationship between stressful life events and problem behaviors and unhealthy outcomes (McKnight et al., 2002; Reyes et al., 2016; Cava et al., 2018; Singhal and Rastogi, 2018; Li et al., 2019).

The results of these studies suggest that life satisfaction is a precursor of healthy outcomes. Life satisfaction can be conceptualized as a cognitive variable serving as a mediator of the link between environmental exposures and healthy behavioral responses (Proctor et al., 2009). Nonetheless, few studies have analyzed the dual role of life satisfaction as a positive outcome in response to perceived social support, yet also as a mediating variable occurring between antecedent variables and positive health outcomes.

In general, the availability of social support is associated with immigrant health and well-being. Nonetheless, Thoits (1982) suggested that the amount of social support received is not the only relevant aspect of social support; rather, the source of social support is also crucial to a positive appraisal of social support. In this regard, the most relevant sources of social support among migrants have been identified as the nuclear family and the extended family. Both can confer positive effects on health and life satisfaction (Ayón and Naddy, 2013; Guo et al., 2019). Another source of social support is friendships, whose influences have been associated with life satisfaction and stress reduction (Klein et al., 1989). However, few studies have differentiated between support from immigrant and native friends, Moreover, among these studies, their results have been contradictory.

Some researchers have suggested that immigrants tend to establish a large number of positive relationships with other immigrants, while establishing a limited number of supportive relationships with the native residents from the host country (García et al., 2002). Other studies have highlighted the more potent beneficial effects of the social support received from native-born community residents (Garciìa-Cid et al., 2017). Some studies have shown an association between social support that is received from members of the immigrant’s culture of origin, with high levels of stress and depression (Falavarjani et al., 2019). In one study, an association was suggested between having friendships with native friends and a decreased sense of discrimination, although the results of this study remain unclear (Domínguez-Fuentes and Hombrados-Mendieta, 2012).

Understanding the differential effects of various forms of social support based on the source that provides it, is most important. Distinguishing between the different sources of social support that occur within a given settlement community can improve our understanding of the effects of these distinct sources on immigrants’ health and well-being. Which sources of social support are most beneficial for international immigrants? This issue may be especially salient among international immigrants, whereby in their new settlement community immigrants can maintain multiple relationships, although these may develop primarily among family and friends. Having trusted people with whom international immigrants can confide their emotions, problems, or difficulties, to experience “being heard and accepted,” has been shown to have a strong effect on the individual’s ability to cope effectively with stressful situations (Lin and Ensel, 1989).

## Sense of Community (Soc), Well-Being and the Health of Immigrant Populations: the Role of the Soc as Mediator

Studies have supported the “concept of community” beyond community when defined by spatial localization. That is, the effects of social networks and social interaction as indicators of “community,” can be more broadly conceived. According to McMillan and Chavis (1986), a “*sense of community* is a feeling that members have of belonging, a feeling that members matter to one another and to the group, and a shared faith that members’ needs will be met through their commitment to be together” (p. 9). Integral to attaining a SOC is a feeling of emotional safety created by membership, and a sense of belonging to and identification with a larger community. They proposed a multidimensional structure for the construct of SOC, as one that consists of four dimensions: needs fulfillment, group membership, influence, and emotional connection.

The process of immigration and adaptation to a new country implies that this major relocation disrupts and diminishes this SOC as linked to one’s culture of origin. This discontinuity also prompts the need to re-establish a new SOC as part of the process of integration into the new cultural environment that exists within a new settlement community (Bathum and Baumann, 2007). In this process, immigrants must also develop new adaptation strategies (Downie et al., 2007). Thus, among international immigrants, this SOC gains special relevance, because it can facilitate social integration into the new settlement community, perhaps also promoting enhanced health and well-being (Albanesi et al., 2007; Cicognani et al., 2008).

The degree of interaction and social integration among and between immigrants and with the rest of the settlement community are core elements for promoting immigrant health and well-being (Foroughi et al., 2001; Jasinskaja-Lahti and Liebkind, 2007; Townley and Kloos, 2011). Low levels of a SOC may compromise health and wellness, from the absence of people with whom to share daily problems, and as this can lead to the development of high stress levels (Antonovsky, 1979). By contrast, this SOC can promote sound mental and physical health through the process of social integration and the establishment of positive relationships that mobilize social support, increase shared resources, and build social and human capital. Furthermore, socially integrated individuals often exhibit better quality in their social interactions and have more diverse support resources from which to draw when coping with stressful situations (Cohen et al., 2000).

In summary, several studies have found significant associations: (a) between SOC with depression and mental illness (Li et al., 2011; Huang et al., 2016; Terry et al., 2019); (b) SOC and good health (Carpiano and Hystad, 2011; Johnson et al., 2011; Anderson et al., 2016); as well as, (c) SOC and life satisfaction (Farrell et al., 2004; Kutek et al., 2011; Huang and Wong, 2014; Ng and Fisher, 2016; Yao et al., 2018; Rugel et al., 2019).

Several studies have also found a positive association between social support and SOC (Vieno et al., 2007; Oh et al., 2014). Most of these studies have found that social support is a good predictor of SOC because this type of support helps individuals to meet their daily needs and fosters relationships with others in the community. Thus, the development of social networks promotes connections between people in the community and strengthens their SOC (Vieno et al., 2007; Huang et al., 2016). Community participation based on supportive relationships fosters connections within that community. This outcome can encourage individuals to access positive social networks, as a means of enhancing their well-being (Dalton et al., 2001).

Theoretically, SOC may function as a mediating factor within this nexus that can be strengthened through social support networks (Pretty et al., 1996). SOC can moderate the negative effects occurring during the immigrant’s adaptation process, although most prior studies have investigated the mediating role of the SOC in other populations. Among military spouses, SOC has been examined as a mediator: (a) between social support and psychological well−being (Wang et al., 2015); (b) between social support and participation in youth−based community organizations, and outcomes relevant to adolescent development (Lardier et al., 2019); (c) between the transgender identity and overall well−being (Barr et al., 2016). Terry et al. (2019) found that SOC is an important mediator of community participation and mental health. Despite the importance of these findings, among immigrants, few studies have been conducted that analyze the mediating effects of SOC on physical health and mental well-being.

## Present Study

As noted, several studies have highlighted social support and the SOC as variables that are positively associated with health and life satisfaction among immigrant populations. However, there is a lack of studies that have concurrently investigated the association between both variables using structural model analysis and the contributing effects of the multi-dimensional factor of social support. As one limitation of prior research, despite the multidimensional nature of social support, researchers have generally used measures that do not distinguish between various sources of immigrant social support. However, other studies have emphasized the relevance of differentiating between sources of support in order to identify those that are most relevant to the welfare of the population under study (Procidiano and Heller, 1983). Furthermore, studies on sources of immigrant support have obtained contradictory results (García et al., 2002; Garciìa-Cid et al., 2017). This aspect is particularly relevant in immigrant populations because social networks can provide resources that are adapted to their needs, also facilitating their social and community integration and their well-being. Therefore, as suggested by ecological and systemic models, it is essential to analyze social support as obtained from different sources and settings (Levitt, 2005). Among immigrants this networking occurs through microsystems, that include family and friends who exist within a specific setting.

There are few studies on the SOC and its associations with SWL, mental health problems, and physical illness in immigrant populations. Thus, the present study investigated SOC as a mediating variable between social support and these outcome variables. By including the SOC and SWL as mediating elements between social support and immigrant health, this study contributes to the establishment of an ecological approach that makes it possible to investigate the person-environment relationship in the host setting by analyzing both interpersonal and contextual factors, as suggested by some authors (Tang et al., 2017).

**FIGURE 1:**
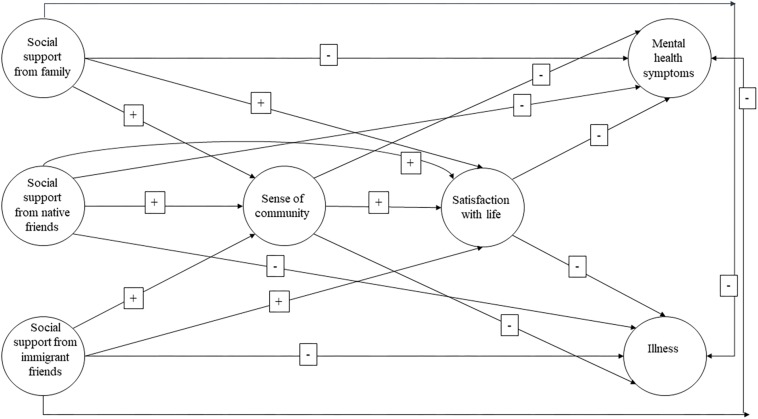
Structural equation model proposed. Path diagram of the theoretical relationships between variables.

We thus propose a model, for an immigrant population, in which perceived social support from the closest sources (family and friends) (see Figure 1) is positively associated with a SOC, and that produce positive health outcomes. This model will test the following general hypothesis: (a) higher scores in the antecedent variable (perceived social support from family and friends) will have a positive effect on the SOC and on SWL, and a negative effect on mental health symptoms and illness.

Specifically, we tested the following hypothesis:

H1: Social support from family and native friends will be positively associated with the SOC.

H2: Social support from family and native friends will be positively associated with SWL and with health.

H3: Social support from family and native friends will have indirect effects on mental health symptoms and illness via their association with higher levels of SOC and SWL.

H4: SOC will be positively associated with SWL, thus associated with a lower level of mental health symptoms and illness.

H5: SWL will be positively associated with mental health symptoms and illness.

In summary, few studies have analyzed the role of different forms of social support and the SOC in immigrant populations, although prior empirical evidence has shown that both variables can have a beneficial effect on well-being. In addition, as examined among immigrants, limited evidence exists from multivariate models that concurrently examine the effects of both factors on SWL and health-related outcomes.

## Materials and Methods

### Participants

The participants consisted of 1131 immigrants from Eastern Europe (Ukraine, Romania, Bulgaria, and Russia), Africa (Maghreb) and Latin America (Paraguay, Argentina, Colombia, and Venezuela), 49% were men and 51% were women. The age range was 18 to 70 years (*M* = 33; *SD* = 12). They had lived in Malaga (Spain) for an average of 8.77 years (*SD* = 6.49) and 53.7% were employed. This distribution is representative of the distribution of immigrants in the city in which this study was conducted, as referenced by the 2017 census data.

### Procedure

This study was conducted in Malaga, which is divided into 11 Municipal Districts. Municipal Districts are large territorial divisions whose boundaries are set by the city council; these divisions are subdivided into neighborhoods. This involved sampling from the 11 Malaga Districts having largest concentration of immigrants.

Data were collected using a *random-route sampling* and survey methodology. The interviewer takes a randomly chosen route from a point of origin in an area and, following the established route, must randomly select the sample units. Boundaries were established for each of the neighborhoods selected, and random route sampling was used to designate the blocks, streets, sidewalks, and so on, in each neighborhood.

Carefully trained interviewers administered the surveys. The questionnaires applied to the non-Spanish-speaking people were translated into their language of origin (i.e., English, French, Russian, and Arabic) by native speakers who had a full command of Spanish. The surveys were conducted in immigrant associations, businesses, meeting places, and Social Service Centers located within each district. All participating immigrants were volunteers and signed an informed consent. No incentives were offered for their participation. The ethical commission of the University of Malaga (CEUMA) determined the suitability of the protocol.

### Measures

#### Mental Health Symptoms

The Spanish version of the Goldberg General Health Questionnaire (GHQ-12) was used (Villa et al., 2013). This questionnaire has been shown to be an effective tool for the evaluation of mental health symptoms in clinical patients and the general population. This questionnaire consists of 12 items. The items are answered on a 4-point Likert-type scale (0–3) ranging from (0) = *Not at all*, to (3) = *Much more than usual* (e.g., “Have you felt constantly overwhelmed and stressed?”). The scale has a Cronbach’s α = 0.817.

#### Satisfaction With Life

The 5-item Satisfaction with Life Scale (SWLS), developed by Pavot and Diener (1993). It was used to assess life satisfaction or the cognitive component of well-being. The items are answered on 7-point scale that ranges from 1 = *completely unsatisfied* to 7 = *completely satisfied* (e.g., “I am satisfied with my life”). The scale has a Cronbach’s α = 0.879.

#### Social Support

Questionnaire on the Frequency of and Satisfaction with Social Support (Garciìa et al., 2016). This instrument measures the frequency of and satisfaction with social support received from the family (family in the host country and the country of origin), immigrant friends (those whom have also immigrated to the new host country), and native friends (those who are native-born residents of the new host country). Social support is divided into three domains: (a) *emotional support* (“Give you love and affection and listen to you when you want to talk and express your feelings”), (b) *instrumental support* (“Willing to do you a favor or do specific things for you; lend you money, accompany you to the doctor, etc.”), and (c) *informational support* (“Give you useful advice and information to resolve doubts, problems, or things you have to do on a daily basis”). We assessed the frequency of and satisfaction with the three types of support (emotional, instrumental, and informational) from each source (family, immigrant friends, and native friends). This instrument consists of 18 items. Frequency of support is measured using a 5-item Likert-type scale (1 = “*Rarely*”, 5 = “*Always*”). Degree of satisfaction with the support received is also measured using a 5-item Likert-type scale (1 = “*Unsatisfied*”, 5 = “*Very satisfied*”). The results of the assessment of the frequency of and satisfaction with the three types of support were used to construct the three indexes (each of which comprised six items) involving different sources of social support (family, and native and immigrant friends). Cronbach’s α was 0.904 for social support from the family, 0.931 for social support from native friends, and 0.94 for social support from immigrant friends.

#### Sense of Community Index SCI-2 (Chavis et al., 2008)

The SCI-2 is a reliable measure. This instrument is based on the SOC model provided by McMillan and Chavis (1986), which assesses need fulfillment, group membership, influence, and emotional connection. This questionnaire consists of 24 items that are measured on a Likert-type scale: (1 = *Not at all*, 2 = *Somewhat*, 3 = *Mostly*, 4 = *Completely*). (e.g., “I get important needs of mine met because I am part of this community”). Based on the recommendations of the authors, the total Sense of Community Index was used (Chavis et al., 2008). The SOC Global Index has a Cronbach’s α = 0.932.

#### Illness Questionnaire (Instituto Nacional de Estadística [INE]., 2017)

This questionnaire consists of a list of 28 illnesses or health problems (hypertension, diabetes, headaches, allergies, etc.). Participants are asked if they have experienced any of them during the last 12 months (“Have you had this problem in the last 12 months?”), if a doctor has diagnosed them (“Has a doctor told you that you have this problem”), and if they needed medication (“Are you taking or have you taken medication for this problem in the past 12 months?”). Based on the answers to the three questions, three indicators were calculated that represent the state of health and any problems (number of illnesses or health problems) that the participants have experienced during the past 12 months. The maximum number of illnesses experienced is 27. Item 26 (prostate problems) excludes women, and item 27 (menopause problems) excludes men. The number of illnesses experienced during the last 12 months ranged from 0 to 15 (mean = 1.97), the number of illnesses diagnosed by a doctor ranged from 0 to 13 (mean = 1.35), and the number of participants who needed medication ranged from 0 to 11 (mean = 1.18).

### Analytical Strategy

Structural Equation Modeling (SEM) path analysis techniques were used. This study consists of a cross-sectional design, and thus examined correlations among variables rather than temporal relationships. This cross-sectional study analyzed the hypothesized associations between the variable domains using the LISREL 9.30 software package (Jöreskog et al., 2016). The model parameters were estimated using the maximum likelihood (ML) method. A structural equation model was used to determine whether there was a positive association between social support from different sources (family, native friends, and immigrant friends) and the SOC, and whether higher levels of SOC will be positively associated with SWL and negatively with mental health symptoms and illness. A latent variable structural equation modeling approach requires the specification of a measurement model as the basis for specifying a structural model. This measurement model was based on the conceptual model presented in Figure 1.

All the measurement items described above were used to calculate a single measurement model, which includes all the latent exogenous and endogenous variables. Regarding the exogenous variables, the observed variables were drawn from the 18 items that assessed the frequency of and satisfaction with emotional, informational, and instrumental support as provided by the three sources of social support. These three sources were used to identify the latent variables. The endogenous latent variables were constructed from the 24 items of the SCI-2, the five items of the SWLS, the 12 items from the questionnaire on mental health symptoms, and three items from the illness questionnaire. Confirmatory factorial analysis was used to test the measurement model. The results of this measurement model were used to calculate the factorial loading scores of the exogenous and endogenous variables.

The proposed structural model consists of three exogenous variables (family support, support from native friends, and support from immigrant friends) and four endogenous variables (the SOC, SWL, mental health symptoms, and illness). Figure 1 shows the network of hypothesized relationships between the latent factors/variables. This model hypothesizes that there will be direct effect pathways from the three sources of social support (exogenous variables/factors) to four variables/factors (endogenous variables: the SOC, SWL, mental health symptoms, and illness). Within the context of this cross-sectional study, a positive association was hypothesized between the sources of social support and SOC and SWL, and a negative association between these sources and mental health symptoms and illness. In addition, it was hypothesized that there would be a direct and positive association between the SOC and SWL and a negative association between the SOC and mental health symptoms and illness. A direct association was hypothesized between SWL and mental health and illness in immigrants. Social support from the three sources was also hypothesized to be positively associated with SWL and negatively associated with health problems via the SOC. The SOC leads to a lower occurrence of health problems via SWL.

We used two models to sequentially test these hypotheses. The first model conceptualizes SOC as a mediating variable between the three predictor variables and SWL, mental health, and illness. This model was used to analyze direct associations of the three sources of social support and the indirect ones via the SOC. In the second model, SWL was added to the SOC as a mediating variable. This approach makes it possible to distinguish the direct effects of the three sources of social support on the two health variables from the indirect effects via SOC and SWL.

## Results

### Measurement Model

Tables 1, 2 show the factor loadings from each measured variable on the latent factor. Table 1 shows each of three latent factors that distinguish different sources of social support. It also shows the mean and standard deviation next to each of the items, the *R*^2^ values of the items — which can be interpreted as reliability indicators (Bollen, 1989) — and the factor loading scores of each item according to their respective dimension of social support. The factor loading scores of the family social support indicators are high (i.e., all scores are around 0.80). The reliability scores of each item are also high (i.e., >0.60). The *R*^2^ values and the factor loading scores of the items corresponding to social support from native friends are higher, and the indexes corresponding to social support from immigrant friends are even higher. These results indicate that each of the latent factors was well identified from its respective measured variables.

**TABLE 1:** Measurement model.

**Items**	***M***	***SD***	***R*^2^**	**SSF**	**SSNF**	**SSIF**
**Social support from family**						
Frequency of emotional support	4.05	1.110	0.668	0.817		
Satisfaction with emotional support	4.16	1.018	0.746	0.864		
Frequency of instrumental support	3.95	1.160	0.610	0.781		
Satisfaction with instrumental support	4.15	1.063	0.698	0.835		
Frequency of informational support	3.97	1.138	0.655	0.810		
Satisfaction with informational support	4.10	1.066	0.748	0.865		
**Social support from native friends**						
Frequency of emotional support	3.58	1.195	0.790		0.889	
Satisfaction with emotional support	3.80	1.139	0.804		0.897	
Frequency of instrumental support	3.27	1.289	0.654		0.809	
Satisfaction with instrumental support	3.59	1.243	0.710		0.843	
Frequency of informational support	3.56	1.212	0.731		0.855	
Satisfaction with informational support	3.77	1.159	0.754		0.868	
**Social support from immigrant friends**						
Frequency of emotional support	3.53	1.252	0.757			0.870
Satisfaction with emotional support	3.72	1.193	0.833			0.913
Frequency of instrumental support	3.25	1.306	0.670			0.819
Satisfaction with instrumental support	3.53	1.272	0.774			0.880
Frequency of informational support	3.44	1.262	0.727			0.853
Satisfaction with informational support	3.62	1.243	0.809			0.900

*Exogenous variables social support from family (SSF), native friends (SSNF), and immigrant friends (SSIF).*

**TABLE 2:** Measurement model.

**Items**	***M***	***SD***	***R*^2^**	**SOC**	**SWL**	**MHS**	**ILL**
**Sense of community**							
My important needs are fulfilled because I am part of this neighborhood.	2.48	0.820	0.422	0.649			
My neighbors and I value the same things.	2.28	0.787	0.384	0.620			
This neighborhood has been successful in meeting the needs of its residents.	2.43	0.745	0.339	0.582			
Being a member of this neighborhood makes me feel good.	2.65	0.810	0.495	0.704			
When I have a problem, I can talk to the neighbors.	2.10	0.906	0.441	0.664			
The people in this neighborhood have similar needs, priorities, and goals.	2.31	0.801	0.331	0.576			
I can trust the people of this neighborhood.	2.25	0.770	0.430	0.656			
I can recognize most of the residents in this neighborhood.	2.55	0.870	0.272	0.522			
Most of the residents in this neighborhood know me.	2.48	0.865	0.274	0.524			
This neighborhood has symbols and expressions such as signs and landmarks that people can recognize.	2.37	0.890	0.178	0.422			
I put a lot of time and effort into this neighborhood.	1.95	0.848	0.423	0.650			
Being a member of this community/neighborhood is a part of my identity.	2.13	0.905	0.561	0.749			
Feeling that I belong to this community/neighborhood is important to me.	2.36	0.882	0.616	0.785			
This community/neighborhood can influence other communities/neighborhoods.	2.10	0.828	0.293	0.541			
I care what other residents in the neighborhood think of me.	2.06	0.957	0.329	0.574			
I have an influence on how the neighborhood is.	1.82	0.847	0.315	0.561			
If there is a problem in this neighborhood, the members can solve it.	2.31	0.768	0.349	0.590			
This neighborhood has good leaders.	2.04	0.867	0.389	0.624			
It is very important for me to be part of this community/neighborhood.	2.33	0.901	0.650	0.806			
I spend a lot of time with other residents of the neighborhood and I really enjoy being with them.	2.07	0.931	0.555	0.745			
I hope to be part of this neighborhood for a long time.	2.57	0.911	0.510	0.714			
The residents of this neighborhood have shared important events together, such as holidays, celebrations, or disasters.	2.32	0.958	0.418	0.647			
I feel hopeful about the future of this neighborhood.	2.49	0.879	0.493	0.702			
The residents of this neighborhood care about each other.	2.28	0.802	0.422	0.649			
**Satisfaction with life**							
In most things, my life is close to my ideal.	4.39	1.382	0.693		0.832		
The conditions of my life are excellent.	4.26	1.423	0.676		0.822		
I am satisfied with my life.	4.72	1.451	0.771		0.878		
So far, I have achieved the things that are important to me in life.	4.71	1.490	0.631		0.794		
If I were to be born again, I would like everything to be the same again in my life.	4.01	1.770	0.446		0.668		
**Mental health symptoms**							
Were you able to concentrate properly on what you were doing?	1.22	0.675	0.124			0.352	
Have your concerns caused you to lose much sleep?	1.13	0.898	0.286			0.535	
Have you felt that you are playing a useful role in life?	1.02	0.671	0.208			0.456	
Have you felt capable of making decisions?	0.93	0.687	0.164			0.404	
Have you felt constantly overwhelmed and stressed?	1.24	0.922	0.376			0.613	
Have you felt that you are unable to overcome your difficulties?	0.98	0.861	0.381			0.617	
Have you been able to enjoy your normal daily activities?	1.07	0.665	0.244			0.494	
Have you been able to address your problems adequately?	1.02	0.593	0.255			0.505	
Have you been feeling unhappy or depressed?	0.86	0.872	0.608			0.780	
Have you lost confidence?	0.55	0.763	0.547			0.740	
Have you felt worthless?	0.39	0.709	0.502			0.709	
Do you feel reasonably happy all things considered?	0.95	0.610	0.258			0.508	
**Illness**							
Number of diseases or health problems. “Have you had this problem in the last 12 months?”	1.97	2.217	0.801				0.895
Number of diseases or health problems. “Has a doctor told you that you have this problem?”	1.35	1.872	0.863				0.929
Number of diseases or health problems. “Are you taking or have you taken medication for this problem in the past 12 months?”	1.18	1.682	0.870				0.933

*Endogenous variables sense of community (SOC), satisfaction with life (SWL), mental health symptoms (MHS), and illness (ILL).*

Table 2 shows the results of the endogenous variables. It shows that all 24 indicators of the SOC had high factor loading scores. In only one case was the score less than 0.50. The *R*^2^ values were also high. SWL is represented by five indicators. The factor loading scores and *R*^2^ values show that the five items had a good fit to this construct. The items of the mental health symptoms dimension obtained lower scores, although in only three cases were they less than 0.40. Finally, the illness dimension obtained very high scores for each of the three indicators used to measure it.

### Descriptive Statistics and Correlations

Table 3 shows the descriptive statistics and correlations between variables. It should be noted that the descriptive variables are the factorial scores derived from the measurement model. A positive correlation was found between the three types of social support and the SOC and SWL. To different degrees, a negative correlation was found between the three sources and mental health symptoms and illness. A negative correlation was found between the SOC and mental problems, and a small positive correlation between the SOC and illness. A negative correlation was found between SWL and the two indicators of health problems.

**TABLE 3:** Descriptive statistics and correlations.

	**SOC**	**SWL**	**MHS**	**ILL**	**SSF**	**SSNF**	**SSIF**
**Sense of community**							
Satisfaction with life	0.303^∗∗^						
Mental health symptoms	–0.100^∗∗^	–0.305^∗∗^					
Illness	0.064^∗^	–0.120^∗∗^	–0.128^∗∗^				
Social support from family	0.150^∗∗^	0.402^∗∗^	–0.228^∗∗^	–0.103^∗∗^			
Social support from native friends	0.244^∗∗^	0.379^∗∗^	–0.141^∗∗^	–0.053	0.477^∗∗^		
Social support from immigrant friends	0.125^∗∗^	0.218^∗∗^	–0.056	–0.083^∗∗^	0.389^∗∗^	0.377^∗∗^	
*M*	1.146	1.349	0.040	1.056	1.020	0.753	0.440
*SD*	0.417	1.262	0.057	1.724	0.705	0.687	0.583

*Variables are the factorial scores derived from the measurement model. Sense of community (SOC), Satisfaction with life (SWL), Mental health symptoms (MHS), Illness (ILL), Social support from family (SSF), Social support from native friends (SSNF), and Social support from immigrant friends (SSIF). ^∗∗^*p* < 0.01, *^∗^p* < 0.05.*

### Structural Model

Table 4 shows the results of the two structural models. The global adjustments and explained variance (*R*^2^) of each of the variable criteria improved from model 1 to model 2. The final values were: NFI = 0.967, CFI = 0.970, IFI = 0.970, GFI = 0.991, AGFI = 0.935, RMR = 0.003, SRMR = 0.031, RMSEA = 0.086, *R*^2^ SOC = 0.062, *R*^2^ SWL = 0.250, *R*^2^ MHS = 0.10, and *R*^2^ ILL = 0.032. This table shows the statistically significant associations (*p* < 0.05).

**TABLE 4:** Unstandardized structural coefficients, standard errors (in brackets) and *t*-values of the direct and indirect relationships between the predictor variables and criterion variables.

		***Model 1 (Mediating variable: SOC)***		***Model 2 (Mediating variables: SOC* + *SWL)***	
**Predictor variable**	**Criterion variable**	**Direct effect**	**Indirect effect**	**Direct effect**	**Indirect effect**
SSF	SOC	0.021 (0.020) 1.05		0.021 (0.020) 1.05	
SSN		**0.131 (0.021) 6.40**		**0.131 (0.021) 6.40**	
SSI		0.021 (0.023) 0.91		0.021 (0.023) 0.91	
SSF	SWL	**0.490 (0.054) 9.02**	0.014 (0.013) 1.05	**0.490 (0.054) 9.02**	0.014 (0.013) 1.05
SSN		**0.351 (0.056) 6.22**	**0.085 (0.017) 5.01**	**0.351 (0.056) 6.22**	**0.085 (0.017) 5.01**
SSI		0.028 (0.062)0.441	0.014 (0.015) 0.90	0.028 (0.062) 0.44	0.014 (0.015) 0.90
SOC		**0.648 (0.081) 8.04**		**0.648 (0.081) 8.04**	
SSF	MH	**−0.018 (0.003) −6.43**	0.000 (0.000) −0.94	**−0.012 (0.003) −4.32**	**−0.006 (0.001) −6.04**
SSN		−0.003 (0.003) −1.18	**−0.001 (0.001) −2.02**	0.001 (0.003) 0.25	**−0.005 (0.001) −5.27**
SSI		0.005 (0.003) 1.61	0.000 (0.000) −0.83	0.005 (0.003) 1.75	−0.001 (0.001) −0.66
SOC		**−0.009 (0.004) −2.12**		−0.001 (0.004) −0.27	**−0.008 (0.001) −5.66**
SWL				**−0.012 (0.001) −7.95**	
SSF	ILL	**−0.220 (0.085) −2.59**	0.008 (0.008) 0.99	−0.136 (0.087) −1.55	**−0.076 (0.026) −2.98**
SSN		−0.025 (0.088) −0.29	**0.047 (0.018) 2.61**	0.035 (0.089) 0.39	−0.013 (0.026) −0.50
SSI		−0.162 (0.097) −1.67	0.008 (0.009) 0.87	−0.158 (0.097) −1.63	0.003 (0.014) 0.20
SOC		**0.359 (0.126) 2.85**		**0.470 (0.129) 3.65**	**−0.111 (0.033) −3.37**
SWL				**−0.171 (0.046) −3.70**	

*In bold the significant coefficients (*p* < 0.05). Social support from family (SSF), Social support from native friends (SSNF), Social support from immigrant friends (SSIF), Sense of community (SOC), Satisfaction with life (SWL), Mental health symptoms (MHS), and Illness (ILL).*

The first model introduced SOC as a mediating variable. The mediating role of this variable can be seen in the indirect effects. A significant positive association was found between family social support and SWL (γ = 0.504) and a negative association between this source and mental health symptoms (γ = −0.018) and illness (γ = 0.213). A positive association was also found between social support from native friends and the SOC (γ = 0.131) and SWL (γ = 0.436). No significant association was found between social support from immigrant friends and the criterion variables. Table 4 shows that the SOC exhibited a small mediating effect. This is shown by the greater positive association between support from native friends and SWL (γ = 0.085). The SOC exhibits a very small mediating effect between support from native friends and mental health symptoms (γ = −0.001). It is noteworthy that the SOC exhibits a small positive effect between support from native friends and illness (γ = 0.047). Table 4 shows a strong association between the SOC and SWL (β = 0.648); however, the SOC exhibited a weaker association with mental health symptoms (β = −0.009) and illness (β = 0.359). The association between the SOC and illness was positive, in contrast to the original hypothesis.

The second model included SWL as the second mediating variable. The association between family support and mental health symptoms was mainly mediated by SWL (γ = −0.006) and not by SOC (γ = −0.000). Similarly, the association between support from native friends and mental health symptoms was mediated by SWL (γ = −0.005) and not by SOC (γ = −0.001). Family support was associated with lower levels of illness via SWL (γ = −0.076) rather than via the SOC (γ = 0.008). Life satisfaction mediated the indirect effect of SOC on mental health symptoms (β = −0.008) and illness (β = −0.111). A direct negative association was found between SWL and mental health symptoms (β = −0.012) and illness (β = −0.171).

**FIGURE 2:**
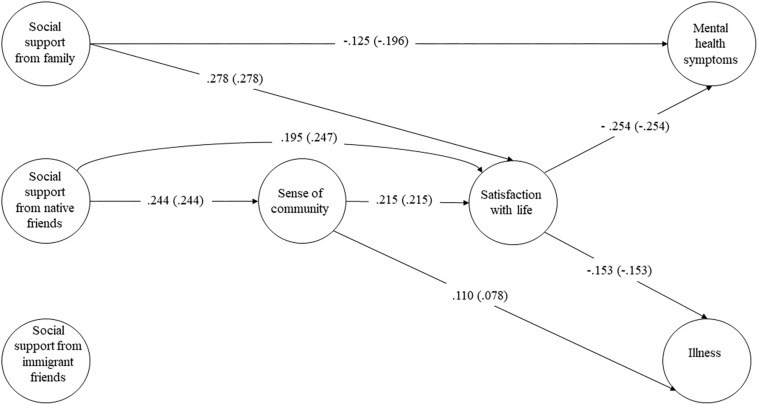
Empirical model of the relationships between the variables. Statistically significant direct coefficients (total effects, in bracket) obtained for the structural equations model proposed.

Finally, the second model was recalculated and paths that were not statistically significant (*t*-value < 1.96, *p* > 0.05) were eliminated. Its global adjustment indexes are good (NFI = 0.955, CFI = 0.966, IFI = 0.966, GFI = 0.987, AGFI = 0.977, RMR = 0.018, SRMR = 0.037, and RMSEA = 0.051). Figure 2 shows the standardized coefficients. Social support from the family and native friends explained greater SOC and SWL, and lower mental health symptoms and illness. No statistically significant association was found between social support from immigrant friends and the remaining variables. The SOC exhibited a small mediating effect, although it was associated with greater SWL, fewer mental health symptoms, and more illness. Life satisfaction was associated with fewer mental symptoms and less illness; it also had capacity to mediate the effect of social support and the SOC.

## Discussion

The main objective of this study was to investigate the association of social support and the SOC with SWL and immigrant health. We proposed a model in which perceived social support from the closest sources, would be as mediated by the SOC and life satisfaction, which in turn would be positively associated with mental and physical health.

Results provide support for many of the hypothesized associations between these variables, and in the predicted direction. Positive associations were found between social support from native friends and higher scores in the SOC and SWL. A positive association was also found between the SOC and SWL. Finally, a strong association was found between SWL and lower levels of the number of mental health symptoms and number of illnesses.

The results provide partial support for H1 and H2. This study design allowed us to analyze in greater depth the complexity of associations between these variables and their effects on SWL and subsequently with mental and physical health. It was important to examine three distinct sources of social support to identify the distinct effect contributed by each of these sources of social support on other factors of interest. We found a positive association between support from native friends (those born in the new host country) and the SOC, and a positive association between support from the family and native friends and greater SWL. On the other hand, no association was found between perceived support from immigrant friends and the SOC or SWL.

The latter result may be explained by the fact that satisfaction with support is mainly determined by the needs of the individuals (Lin, 1986). This aspect is included in the Theory of Specificity by Cohen and McKay (1984), who suggested that social support is more effective when it is adjusted to the problem that needs to be addressed. It may be the case that positive interactions with native members of the community would better meet the needs of immigrants in the host society. In other words, among immigrants, a SOC best develops from social support that is received from native residents from the host country. It has been found that psychosocial adjustment among this group of international immigrants is facilitated by the presence of native people in their social networks, and by satisfaction with the contact maintained with members of the host society (Searle and Ward, 1990). It could also be the case that native-born individuals, those from the host country, have more to offer in terms of social support. They may have more social and human capital to offer the immigrant, since they are well acquainted with the customs and practices of the host society, as compared with immigrants who are still trying to acculturate and to understand the customs and practices of the new host society.

In addition, other studies have shown that although immigrants can have access to different sources of support, in general they experience significant disadvantages when they receive support from others belonging to their own ethnic group, who often have less social capital and limited resources that they can offer the immigrant person (Kim and McKenry, 1998). On the other hand, positive relationships with individuals from the new host society may make the immigrant feel more integrated and accepted within the new the host country, and lead to an increase in perceived support and life satisfaction (Domínguez-Fuentes and Hombrados-Mendieta, 2012). Conversely, among immigrants, this effect also suggests the potent and detrimental effects of discrimination when it is received from persons who are natives of the host society.

These results also allow us to analyze the support needs of immigrants from a dynamic perspective. Support needs change as the migration process evolves (Kahn and Antonucci, 1980). When immigrants first arrive in a new country they seek contact, support, and validation from their compatriots, but as they settle down and integrate into the host country they seek contact, support, and validation from members of the new host society. From this perspective, it would be of interest to conduct an in-depth qualitative interview analysis of contextual factors in social support from immigrant friends, which we found to have the weakest associations as influences on SOC and SWL.

In general, many studies have found an association between perceived support and mental health symptoms and illness (Kristiansen et al., 2006; Dinh et al., 2009; Jibeen, 2011). In general, social relationships typically exert a positive effect on immigrant health such that social support from family and friends may serve as indicators of optimal levels of health (Zhang and Ta, 2009). The results of our study show that family support has the strongest effect on lower levels of mental health symptoms. In this model, perceived family support is the best protector of the mental health of immigrants. This result is consistent with studies that have shown that family relationships help immigrants to maintain appropriate health behaviors, and to avoid specific risk behaviors, to improve their quality of life (Gracie et al., 2012; Aqtash and Van Servellen, 2013).

These findings are also corroborated by other studies that have suggested that family support is the type of support associated with fewer mental health symptoms and more positive well-being (Runyan et al., 1998). Moreover, the health of immigrants is significantly compromised by negative experiences, including discrimination experience within the host country, although there exist remarkable differences involving family support and integration in the community (McClure et al., 2015). Results of the present study confirm the need to examine the different sources of support to better understand the actual experience of support. This approach is suggested by ecological and systemic models (Bronfenbrenner, 2005) that emphasize the relevance of the development of social relationships through key microsystems such as family and friends.

The results of this study partly support H3, given that the SOC exhibited a small mediating effect. On the other hand, SWL exhibited a stronger mediating effect than the SOC. This result is of interest and demonstrates the relevance of SWL within an immigrant population. In fact, few studies have analyzed the dual role of life satisfaction as a beneficial outcome of perceived support and as a mediating variable between the variables that precede it and positive health outcomes. Some authors have suggested that SWL should be considered as a significant intervening cognitive variable (Proctor et al., 2009).

These study results partly support H4. A strong association was found between the SOC and SWL. For international immigrants, this result suggests the beneficial effects of successful integration into a new host community on immigrants’ SWL. A significant positive association was found between the SOC in immigrant populations and their SWL. Some studies have highlighted the association between SOC and the SWL (Fugl-Meyer et al., 2002; Hombrados-Mendieta et al., 2009; Moscato et al., 2014). However, although no association was found between the SOC and mental health symptoms, a positive association was found between the SOC and illness. This finding may be explained by the fact that controlling interaction at the community level could be more difficult than at the interpersonal level, which may lead to more stress when the possibility of controlling contact is absent (Altman, 1975).

Another explanation is that increased contact and interaction with others may increase the risk of contagion of other illnesses. This aspect may be particularly relevant for immigrant populations, which must adapt to new illnesses within the host society. Malgesini (2002) pointed out that the most common illnesses are reactive illness, adaptive illnesses, or illnesses acquired in the community due the working conditions experienced, as well as in their living conditions, that can include overcrowding and poverty, which increase the risk of members of an immigrant community contracting certain illnesses.

Furthermore, integration into a new community is often contingent on acquiring the new customs, habits and lifestyles of members from the host society. Among international immigrants, this assimilation may occur with changes that involve the acquisition of unhealthy behavioral patterns, or with the acquisition of healthier behaviors. This process can depend on the immigrant’s particular reference group (Gordon-Larsen et al., 2003). Latino immigrants, especially Mexicans who migrate to the US, are a case in point. With greater length of residency and acculturation into the mainstream society, many experience an increase in health problems, such as diabetes, because they abandon the habits of their country of origin, adopting less healthy habits, and consuming products that are inexpensive and readily available.

The SOC corresponds to a deeper level of analysis and it should be analyzed to determine whether community resources satisfy the needs of the immigrant population. To promote the health of immigrants, it would be useful to develop resources that satisfy health needs in order to strengthen the association between the SOC and improved health (Hombrados-Mendieta et al., 2013a). In fact, some studies have shown that immigrant populations experience difficulties in accessing mental health services and make less use of them (Gilgen et al., 2005; Nadeem et al., 2008; Tieu and Konnert, 2014).

The results of this study also support H5. Significant associations were found between SWL and a lower scores in mental health symptoms and illness. When immigrants feel satisfied with life in the community, there is a negative association between SWL and mental health symptoms and illness. According to the model, SWL depends on having support networks, mainly family and native, and being integrated into the community.

In summary, this study has demonstrated an important association between the SWL of immigrants and their physical and mental health. Other studies have obtained similar results (Moreta-Herrera et al., 2018).

### Limitation of This Study

This study has a few limitations. It should be noted that results related to the migration processes are strongly influenced by the setting in which a cohort of immigrants live (Long and Perkins, 2007). Accordingly, generalizations arising from these types of studies should be conducted with caution. This study consisted of a cross-sectional design, and accordingly, the temporal sequence of events depicted in our model is suggestive, and not confirmatory. We recognize that explanations involving a reversal in temporal ordering in our model would also be plausible. In a future study, it would be of interest to analyze all the variables over time to observe actual temporal effects in how the SOC develops, how social networks are created, and how they influence the wellbeing of immigrant populations. Future studies should also identify the elements that facilitate the development of the SOC and SWL in each of several diverse immigrant groups, and that analyze differences as moderated by sociodemographic characteristics (e.g., age, sex, and ethnicity).

### Implications for Research, Practice and Policy

The results of this study are relevant to research, policy, and practice. We have indicated the need to analyze the migration process from a multidimensional approach to social support. We consider it important to design intervention strategies that will facilitate intercultural relationships between immigrants and natives of the host society. In addition, we suggest that positive interaction patterns should be promoted between immigrants and their friends and family, to build positive perceptions of social support. Special attention should be given to immigrants who lack family support. Thus, given that for large periods of time immigrants are geographically separated from their families, health care professionals should work with immigrants to aid in the development of their online social networks (Kogstad et al., 2013).

The present study findings suggest the need to create policies and interventions that encourage the participation of immigrants in relevant community activities, to increase their integration in a community and to develop their SOC. We have shown the importance of studying community resources to analyze whether they actually meet the needs of the immigrant population. A fulfillment of these needs could strengthen the relationship of SOC on health and well-being among members of an immigrant group. This study highlights the importance of health professionals in developing specific tasks that promote community health and disease prevention among immigrants, the promotion of healthy habits, a protection against endemic diseases, and the avoidance of unhealthy behaviors within the new host community (Cunningham et al., 2008). The findings of the present study can also inform the development of strategies to prevent social conflict, improve quality of life, and facilitate intercultural coexistence.

## Conclusion

This study was conducted using a large and diverse sample of international immigrants. Accordingly, these study results may be applicable to similar populations, with implications for the design of health promotion interventions that are applicable within multicultural communities. Furthermore, these results can inform social policies that advocate for the creation of social support networks, positive interactions with individuals from the host community, and that promote immigrants’ effective integration into a local community. The principal aim in these efforts is to improve the health of immigrants, and their health and life satisfaction, as these outcomes can enhance intercultural relations among diverse sectors of a community.

## Ethics Statement

In conducting the study, accepted principles of ethical and professional conduct have been followed (Reference number: CEUMA: 37-2016-H). We obtained ethical approval for the research from the ethics committee of the University of Málaga. All subjects gave written informed consent in accordance with the Declaration of Helsinki. The protocol was approved by ethics committee of the University of Málaga.

## Author Contributions

IH-M, MM-F, and LG-J contributed to conception or design of the work, analyzed the data, and wrote the manuscript. FG-C analyzed the data, substantial contributions to revising the work critically, and wrote the manuscript. MM-M analyzed the data and wrote the manuscript. AG-C collected the data and wrote the manuscript. All authors involved in data analysis and interpretation, approved the final version of the manuscript to be published.

## Conflict of Interest

The authors declare that the research was conducted in the absence of any commercial or financial relationships that could be construed as a potential conflict of interest.
